# The Resveratrol Rice DJ526 Callus Significantly Increases the Lifespan of *Drosophila* (Resveratrol Rice DJ526 Callus for Longevity)

**DOI:** 10.3390/nu11050983

**Published:** 2019-04-29

**Authors:** Mousumee Khan, Soyeon Park, Hyeon-Jin Kim, Kui-Jae Lee, Dea Heon Kim, So-Hyeon Baek, Seong-Tshool Hong

**Affiliations:** 1 Department of Biomedical Sciences and Institute for Medical Science, Chonbuk National University Medical School, Jeonju, Chonbuk 54907, Korea; mou.ku.es@gmail.com (M.K.); microvac_love@naver.com (S.P.); 2 JINIS BDRD Institute, JINIS Biopharmaceuticals Co., 913 Gwahak-Ro, Bongdong, Wanju, Chonbuk 55321, Korea; hjkim@jinisbio.com; 3 Division of Biotechnology, Advanced Institute of Environment and Biosciences, College of Agriculture and Life Sciences, Chonbuk National University, Iksan, Chonbuk 54596, Korea; leekj@jbnu.ac.kr; 4 Department of Biology, Sunchon National University, Suncheon, Jeonnam 57922, Korea; dheonkim@sunchon.ac.kr; 5 Department of Well-being Resources, Sunchon National University, Suncheon, Jeonnam 57922, Korea

**Keywords:** resveratrol, antiaging, lifespan, resveratrol rice DJ526, callus

## Abstract

Resveratrol has gained widespread scientific attention due to its ability to significantly extend the lifespan of yeast. However, research on the efficacy of resveratrol on lifespan extension has yielded mixed results in animal studies, making resveratrol a contentious subject. In our previous work, we reported that transgenic resveratrol rice DJ526 showed unusual health benefits beyond expectations. In this work, we established a callus culture of resveratrol rice DJ526, which contains 180 times more resveratrol than the grain, and found that resveratrol rice callus significantly extended the median lifespan of *Drosophila melanogaster* by up to 50% compared to the control. The resveratrol rice callus also ameliorated age-dependent symptoms, including locomotive deterioration, body weight gain, eye degeneration, and neurodegeneration of *D. melanogaster* with age progression. Considering that resveratrol is the most preferred antiaging compound due to its superior safety and proven mechanism against many serious adult diseases, the outstanding efficacy of resveratrol on the longevity of wild-type animals could cast a light on the development of antiaging therapeutic agents.

## 1. Introduction

Aging is an inevitable biological process characterized by a gradual deterioration in physiological function [1,2], which leads to an increased risk of diseases and, ultimately, to death [3]. Undoubtedly, the aging process poses a great risk for most human diseases [4]. Therefore, it is not surprising that one of the main aims of modern research is to develop a safe therapeutic agent that has beneficial effects for health and antiaging, especially age-related deteriorations and diseases. To date, many pro-longevity compounds have been identified, including resveratrol [5], rapamycin [6], metformin [7], spermidine [8], etc. Among them, resveratrol has been studied most extensively for its effect on longevity due to its safety as well as its beneficial effects against human diseases such as cancer, cardiovascular disease, and neurodegenerative diseases [9].

Resveratrol (3,5,4′-trihydroxy-trans-stilbene) is a non-flavonoid polyphenol-type phytonutrient found in several plant roots and fruits, including the skin of peanuts, blueberries, mulberries, grapes, and raspberries [9]. Although resveratrol is well known to have numerous beneficial health effects, its main function is antiaging [10,11]. The antiaging effect of resveratrol was first noticed in the yeast *Saccharomyces cerevisiae*, in which the lifespan was extended by up to 70% by stimulating *Sir* (silent information regulator) [12]. The antiaging effect of resveratrol in higher organisms, however, is still ambiguous. Studies have shown that resveratrol failed to significantly extend the lifespan of *Caenorhabditis elegans* [13,14]. In *Drosophila melanogaster*, resveratrol extended the maximum lifespan by up to 20% in one study, but other studies failed to produce any significant results [15]. Studies in short-lived fish species have reported the extension of lifespan in *Nothobranchius furzeri* and *N. guentheri* [16,17,18] but not in *Daphnia pulex* [19]. In mammals, there is no substantial evidence suggesting lifespan extension by resveratrol in healthy, wild-type mammals, although marginal increase in lifespan was found in metabolically compromised mammals [20]. In a mice model, resveratrol treatment improved the physiology and reduced the risk of death in mice on a high-calorie diet compared to mice on a standard diet [20]. Resveratrol has also been reported to delay physiological deterioration in mice with age progression by mimicking transcriptional changes as well as the gene expression patterns, although it failed to extend the lifespan [21]. Thus, it can be concluded from the aforementioned studies that the ability of resveratrol to promote longevity is conserved in various organisms along with dietary restriction, but significant extension of lifespan without dietary restriction has not been achieved in healthy, wild-type animals.

In our previous work, we created resveratrol rice, named as DJ526, by transferring the resveratrol biosynthesis gene (stilbene synthase) from the peanut *Arachis hypogaea* variety Palkwang into *Oryza sativa* japonica variety Dongjin (DJ) rice [22,23,24,25]. The resveratrol rice DJ526, which has a significant amount of resveratrol (1.4–1.9 μg/g) in its grain, exhibited unexpectedly high therapeutic effects on metabolic syndrome and obesity in animal studies compared to the resveratrol-fed group or the parental DJ rice-fed group with marginal efficacy [22,23,24,25]. The therapeutic efficacies of the resveratrol rice DJ526, presumably by the synergistic effect of resveratrol with the endogenous property of DJ rice, on metabolic syndrome and obesity were comparable to pharmaceutical drugs aimed at treating these diseases [22,23,24].

Considering the fact that the intrinsic property of resveratrol is antiaging, it would be interesting to investigate whether the synergistic interaction in the resveratrol rice DJ526 could extend the intrinsic property of resveratrol so that the lifespan of animals can be extended to a degree observed in single-celled yeast. To investigate the effect of the synergistic interaction of resveratrol with the innate property of resveratrol rice DJ526 on the extension of animal lifespan, we established a callus culture that contains 180 times more resveratrol than that in the resveratrol rice DJ526 itself. Results show that the resveratrol rice callus dramatically extended the lifespan of *D. melanogaster* by up to 42% compared to the control, while supplementation of the same amount of resveratrol or parental rice callus failed to extend the lifespan of *D. melanogaster*.

## 2. Materials and Methods

### 2.1. Fly Stocks and Maintenance

The wild-type Harwich strain (FBst0004264, stock number 4264) was obtained from the Bloomington Drosophila Stock Center (Indiana University, Bloomington, United States of America USA), and wild-type ORR strain was provided by Dr. R. G. Silva-Oliveira (Laboratory for Cytogenetics and Mutagenesis, Patos de Minas, Brazil). All experimental flies were maintained under a temperature-controlled incubator with a 12 h light/12 h dark cycle at 18 °C and 60% humidity in standard vials containing media with the addition of small amounts of plain food. 

### 2.2. Chemicals and Reagents

The suppliers and catalog numbers for all purchased reagents are as follows. Cornmeal (DFC-30102), active dry yeast (HS-62-103), agar (DFY-30301), molasses (DFM-30701), and *Drosophila* instant food plain (DIFP-31020) were from Hansol (Seoul, Korea). Sucrose (7501-4400) and ethyl alcohol 94.5% (4204-4400) were from Daejung (Siheung, Korea). Resveratrol (sc-200808A) was from Santa Cruz Biotechnology, Inc. (Dallas, TX, USA). Propionic acid (64655-0450) was from Junsei (Tokyo, Japan). Methyl 4-hydroxybenzoate (H3647) and formalin solution 10% (314-771-5765) were from Sigma-Aldrich (St. Louis, MO, USA).

### 2.3. Callus Culture

Brown rice seeds were sterilized with 70% (*v*/*v*) ethanol for 1 min and 2% sodium hypochlorite for 60 min. The seeds were then washed with sterilized distilled water at least 5 times. For callus induction, the seeds were inoculated in 2N6 medium (3.95 g/L N6 salt, 30 g/L sucrose, 1 g/L casamino acid, 2 mg/L 2,4-D, 2 g/L phytagel, pH 5.6) in petri dishes and incubated at 25 °C in the dark for 3 weeks. The optimal culture condition was established by adjusting the physical culture parameters in 2MS-NO_3_ free liquid medium (2.65 g/L MS NH_4_NO_3_ free salt, 30 g/L sucrose, 1 g/L casamino acid, 2 mg/L 2,4-D, pH 5.6). The maximum amount of resveratrol from the callus was achieved from 2MS-NO_3_ free liquid medium with 10-day culture in 1000 mL flasks at 25 °C/light intensity of 2000 Lux/shaking at 100 rpm.

### 2.4. Liquid Chromatography

Agilent 6410B Triple Quadrupole LC/MS (Agilent Technologies, Wilmington, NC, USA) equipped with an electrospray ionization (ESI) source was employed for the analysis. Resveratrol (sc-200808A) was purchased from Santa Cruz Biotechnology, Inc. (Dallas, TX, USA) and used as reference standard. One hundred milligrams of each sample was mixed with 1 mL of methanol for centrifugation, and the supernatants were used for HPLC. Five microliters of the processed samples were injected into the HPLC system (1200 Series LC, Agilent Technologies, Wilmington, USA) fitted with Phenomenex Synergi Hydro-RP 2.6 µm 100 Å 50 × 2.1 mm column, maintained at 30 °C. ESI was operated at +3000 V with a source temperature of 380 °C. Capillary voltage, cone voltage, and source offset were set at 3 kV, 30 kV, and 30 V, respectively. The gas flow of desolvation was set at 650 L/h, 150 L/h with a nebulizer pressure of 15 bar. A mobile phase composed of 0.1% formic acid in distilled water (Buffer A) and 0.1% formic acid in acetonitrile (Buffer B) was used to separate the analytes and pumped into the ESI chamber at a flow rate of 0.5 mL/min for 20 min. Fragmentor voltage and collision voltage was set at 70 V. Detection of the ions was carried out in the multiple-reaction monitoring mode (MRM) by monitoring the transition pairs of m/z 229.1 to 135.1 (resveratrol). Data acquisition was performed with the MassHunter Software (Version B.04.00).

### 2.5. Lifespan Assay

*D. melanogaster* flies were routinely maintained into the following groups for laying eggs: standard cornmeal media (Ctrl), cornmeal supplemented with resveratrol media (RES), DJ callus media (DJ), and the resveratrol rice DJ526 media (DJ526). The compositions of these media are given in Supplementary Table S1. The resveratrol concentration in DJ526 callus media was quantified by LC/MS analysis. The fly larvae were cultured at 25 °C until eclosion. The eclosed flies were collected under light CO_2_ anesthesia within a few hours of eclosion and kept at 25 °C for 48 h for maturation. The matured flies were then transferred into the respective diets as indicated above and raised under a temperature-controlled incubator with a 12 h light/12 h dark cycle at 18 °C and 60% humidity. A total of 300 flies—150 adult male and 150 adult female—were used for the survival assay, and each vial contained 50 flies to avoid crowding. The flies were transferred to new vials with fresh media every 4 days, and the number of dead flies was then recorded. This process was continued until all flies were dead. Individual flies that died of a non-age-related cause, such as getting stuck on food, were censored from the result. Survivability was analyzed using the Kaplan–Meier method. The Kaplan–Meier estimate is the nonparametric maximum likelihood of survival at a given time point *S*(*t*):

(1) $$S(t)=\underset{t_{i}<t}{\Pi }\frac{n_{i}-d_{i}}{n_{i}}$$

where *n_i_* is the number of survivors less the number of censored cases, and *d_i_* is the number of deaths at time point *t_i_* [26].

### 2.6. Startle-Induced Negative Geotaxis Assay

Startle-induced negative geotaxis assay was carried out on the 10th, 30th, 60th, and 90th day. At each experimental time point, 15 flies per group were sedated with CO_2_ and placed in 15 mL conical tube (Cat: 50015), which was previously marked at heights of 10 mL and 2 mL with a highlighter. The top of the tube was removed and closed with cotton wool. The experimental flies were allowed to recover for 30 min prior to assay. A digital camera was replaced in front of the tube, at a distance of 20–30 cm from the tube, to record the movement of flies during the experiment. The “movie” option of the camera was selected, and the conical tube was then tapped gently for a few seconds (e.g., 10 s) to gather the flies at the bottom of the tube. The flies were allowed to climb for 45 s to observe their climbing ability. After 45 s, the video was stopped. The number of flies at the top (above the 10 mL line) and at the bottom (below the 2 mL line) were counted by visual inspection from the recorded video. Three trials were performed for each diet at each time point. The performance index (PI) was calculated for each group of 15 flies (average of 3 trials) using the formula PI = 0.5 × (*n_total_* + *n_top_* − *n_bottom_*)/*n_total_*, where *n_total_* is the total number of flies taken, *n_top_* is the total number of flies at the top, and *n_bottom_* is the total number of flies at the bottom [27].

### 2.7. Body Weight Measurement

Fifteen adult flies per group were anesthetized on a CO_2_ plate and weighed immediately using a Sartorius Electronic weighing balance (Germany). The body weights were measured on the 10th, 30th, 60th, and 90th day. For each group, three independent replicates were averaged.

### 2.8. Eye Imaging with Light Microscope

For light microscope imaging of the eyes, 20 adult flies (10 male and 10 female) from each respective media were collected on the 10th, 30th, 60th, and 90th day and kept in −80 °C temperature for 1 h before taking images. Eye images were taken by AmScope 6.7X to 45X Boom Stereo Dissecting Microscope, equipped with AmScope Microscope Eyepiece Camera (MU1000, AmScope, China).

### 2.9. Histology

Adult male and female flies were collected from each respective media on the 10th, 30th, 60th, and 90th day. Flies were anesthetized with CO_2_ and frozen at −80 °C for 1 h before dissection. Fly heads were then dissected and immediately kept in 10% neutral-buffered formalin overnight. They were processed into paraffin using standard histological procedures. Embedded heads were sectioned at 6 µm and stained with hematoxylin and eosin (H&E). The stained brain images were observed in Apero ScanScope FL (Leica Biosystems, Nussloch, Germany) as well as Phase Contrast Microscope (T690C-PCT200INF-PL, AmScope, China), equipped with AmScope Digital Camera (MU1803, AmScope, China), scale bar, 200 µm.

### 2.10. Scoring of Neurodegeneration

Neurodegeneration was identified by the appearance of vacuolar lesions in the brain tissue. Brain tissues were analyzed after histological examination and quantified. A higher number of lesions in the brain tissues indicated more severe neurodegeneration. The numbers of vacuolar lesions are shown in Figure S2. The scoring was done blind with respect to the four diets or groups used in this study. Six levels of neurodegeneration (0, 1, 2, 3, 4, and 5) were used for quantification (Figure S3).

### 2.11. Statistical Analysis

**Figure 1:**
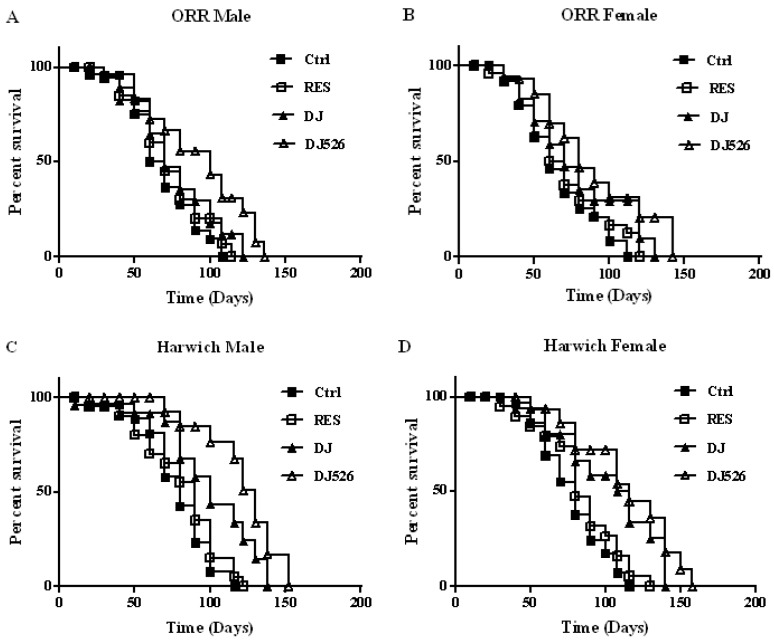
The resveratrol rice DJ526 callus extended the lifespan of *Drosophila melanogaster*. (**A**) ORR male, (**B**) ORR female, (**C**) Harwich male, and (**D**) Harwich female. Ctrl indicates the standard cornmeal media of *Drosophila*; RES (resveratrol) indicates the standard cornmeal diet supplemented with 5692.42 µg/L resveratrol, the equivalent amount of resveratrol content in DJ526 media; DJ indicates the diet in which 50% of cornmeal was replaced with the Dongjin callus; and DJ526 indicates the diet in which 50% of cornmeal was replaced with the DJ526 callus (Table S1). All comparisons were made using Log-rank tests. *p* values (Log-rank tests) for each strain and each sex were as follows: (**A**) ORR male flies: DJ526 versus Ctrl (*p* = 0.0064), DJ526 versus RES (*p* = 0.0284), DJ526 versus DJ (*p* = 0.0803), Ctrl versus RES (*p* = 0.4824). (**B**) ORR female flies: DJ526 versus Ctrl (*p* = 0.0236), DJ526 versus RES (*p* = 0.0656), DJ526 versus DJ (*p* = 0.3446), Ctrl versus RES (*p* = 0.4380). (**C**) Harwich male flies: DJ526 versus Ctrl (*p* < 0.0001), DJ526 versus RES (*p* < 0.0001), DJ526 versus DJ (*p* = 0.0375), Ctrl versus RES (*p* = 0.3857). (**D**) Harwich female flies: DJ526 versus Ctrl (*p* = 0.0002), DJ526 versus RES (*p* = 0.0033), DJ526 versus DJ (*p* = 0.2959), Ctrl versus RES (*p* = 0.2610). Percentage survival is shown along with the maximum lifespan in each group. For the lifespan assays, the survival rate of 150 flies from each group was observed with media changed every 4 days until all flies were dead.

Analysis of survival data was performed using the Kaplan–Meier method, and data was prepared using GraphPad Software Prism version 7.04. All comparisons were made using Log-rank tests (Figure 1). Descriptive statistics are expressed as mean ± standard deviation (s.d.). The statistical differences between two groups were analyzed using an unpaired Student’s *t*-test. All differences were considered statistically significant if *p* values were less than 0.05. The statistical significance is shown as * *p* < 0.05, ** *p* < 0.01, and *** *p* < 0.001 from three independent experiments.

## 3. Results

### 3.1. Resveratrol Rice DJ526C Producing a High Level of Resveratrol Was Obtained

The resveratrol rice callus was induced using the 2N6 callus induction medium from the mature seeds of DJ and DJ526 rice after three weeks of inoculation (Figure S1A). After induction of the callus, the physical culture parameters of temperature, aeration, and light intensity were adjusted to establish culture condition producing maximal amount of resveratrol in callus. The chemical profile of metabolites was analyzed using liquid chromatography (Figure S1B–D). The chemical profiles of the induced callus were analyzed for resveratrol compounds, resveratrol, and resveratrol glucoside (piceid). In the DJ callus, liquid chromatographic analysis failed to detect resveratrol or piceid (Figure S1C), while 2.13 ± 0.02 μg/g of resveratrol and 56.71 ± 0.13 μg/g of piceid were measured in the DJ526 callus (Figure S1D). The maximal amount of resveratrol was produced by culturing the resveratrol rice calli in 2MS-NO_3_ free liquid medium (2.65 g/L MS NH_4_NO_3_ free salt, 30 g/L sucrose, 1 g/L casamino acid, 2 mg/L 2,4-D, pH 5.6) at 25 °C and light intensity of 2000 Lux with 100 rpm shaking for 10 days.

### 3.2. The Resveratrol Rice DJ526 Callus Significantly Extended the Median Lifespan of D. melanogaster

The effect of the resveratrol rice DJ526 callus on lifespan extension was evaluated using two different wild-type strains of *D. melanogaster*: ORR and Harwich. The experiments were conducted by feeding *D. melanogaster* with four different types of diet: control diet (the standard cornmeal diet of *Drosophila*), DJ526 diet (50% of cornmeal in the standard cornmeal diet replaced with DJ526 callus), DJ diet (50% of cornmeal in the standard cornmeal diet replaced with DJ callus), and RES diet (resveratrol, the equivalent amount to the resveratrol rice DJ526 callus, added to the standard cornmeal diet) (Table S1). The efficacy of the resveratrol rice DJ526 callus on the lifespan of *D. melanogaster* was evaluated by calculating dead flies of each group with age progression (Figure 1). The survival rate differences were observed starting from the 30th day of the feeding experiment with ORR flies. The median lifespans of the ORR flies of the DJ526 group were 100 days for male and 80 days for female, which were higher than that of the DJ rice group (70 days for male and 70 days for female), the resveratrol group (70 days for male and 65 days for female), and the control group (70 days for male and 60 days for female). We found that the resveratrol rice DJ526 callus significantly extended the median lifespan of ORR flies compared to the control group (*p* = 0.0064 for male and *p* = 0.0236 for female) and RES group (*p* = 0.0284 for male only) (Figure 1A,B). A similar extension of median lifespan in the DJ526 group was also observed with Harwich, another wild-type fly. The median lifespans of the Harwich flies of the DJ526 group were 130 days for male and 116 days for female, which were higher than that of the DJ group (100 days for male and 116 for female), the RS group (90 days for male and 80 days for female), and the control group (80 days for both male and female). We found that the resveratrol rice DJ526 callus also significantly extended the median lifespan of Harwich flies compared to the control group (*p* < 0.0001 for male and *p* = 0.0002 for female), RES group (*p* < 0.0001 for male and *p* = 0.0033 for female), and DJ group (*p* = 0.0375 for male only) (Figure 1C,D). In addition to the extension of median lifespan, the maximum lifespan of the DJ526 group increased compared to the DJ rice, resveratrol, and control groups (Figure 1). Overall, the resveratrol rice DJ526 callus significantly extended the median lifespan of all tested wild-type flies—ORR male, ORR female, Harwich male, and Harwich female—by 30%, 20%, 50%, and 36%, respectively, compared to the control (Figure 1). These results clearly indicated that the synergistic effect of the transgenic resveratrol in the callus of resveratrol rice DJ526 significantly extended the median lifespan of wild-type *D. melanogaster*.

### 3.3. The Resveratrol Rice DJ526 Callus Ameliorated the Locomotor Deterioration of D. melanogaster during Age Progression

**Figure 2:**
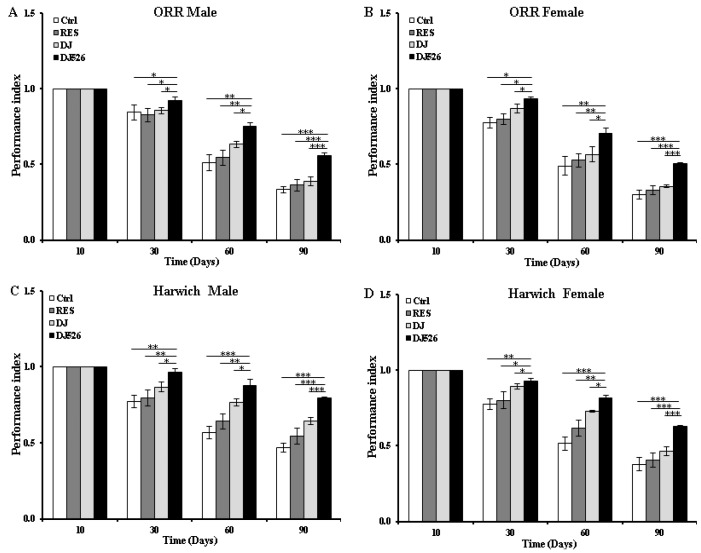
The resveratrol rice DJ526 callus ameliorated the locomotor deterioration of *D. melanogaster*. (**A**) ORR male, (**B**) ORR female, (**C**) Harwich male, and (**D**) Harwich female. Ctrl indicates the standard cornmeal diet of *Drosophila*; RES (resveratrol) indicates the standard cornmeal diet supplemented with 5692.42 µg/L resveratrol, the equivalent amount of resveratrol content in DJ526 media; DJ indicates the diet in which 50% of cornmeal was replaced with the Dongjin callus; and DJ526 indicates the diet in which 50% of cornmeal was replaced with the DJ526 callus (Table S1). The locomotor activity was observed on the 10th, 30th, 60th, and 90th day and indicated as performance index. The data are from three independent experiments, and values are shown as mean ± s.d. An unpaired Student’s *t*-test was used for the statistical analysis; *n* = 15, * *p* < 0.05, ** *p* < 0.01, *** *p* < 0.001.

Behavioral locomotion assay offers an accurate way of assessing muscle function. Considering that the paucity of locomotion is an important indicator of aging [28], we analyzed the locomotive ability of *D. melanogaster* by measuring the climbing ability with age progression (Figure 2). All of the flies in the control group showed a continuous decline of locomotor activities with age progression. However, the locomotor activities of both DJ526-fed ORR and Harwich flies exhibited statistically significant high values compared to other groups starting from the 30th day. Notably, on the 90th day, the DJ526-fed ORR male and female flies climbed the test tube 1.69 and 1.70 times faster than the control group, respectively, which was the best performance compared to the RES group (1.55 and 1.54 times in male and female) and DJ group (1.43 and 1.41 times in male and female). Similarly, the DJ526-fed Harwich male and female flies climbed 1.70 and 1.65 times faster than the control group, respectively, which was the best performance compared to the RES group (1.48 and 1.57 times in male and female) and DJ group (1.25 and 1.36 times in male and female). These results showed that the resveratrol rice DJ526 callus significantly ameliorated locomotive deterioration during age progression, which is consistent with the lifespan increment data (Figure 1).

### 3.4. The Resveratrol Rice DJ526 Callus Maintained a Healthy Body Weight of D. melanogaster during Age Progression

**Figure 3:**
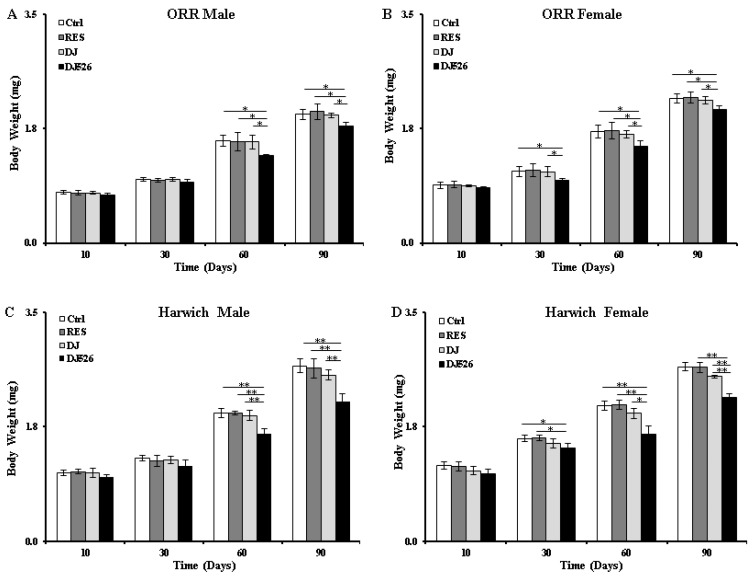
The resveratrol rice DJ526 callus maintained a healthy body weight of *D. melanogaster*. (**A**) ORR male, (**B**) ORR female, (**C**) Harwich male, and (**D**) Harwich female. Ctrl indicates the standard cornmeal diet of *Drosophila*; RES (resveratrol) indicates the standard cornmeal diet supplemented with 5692.42 µg/L resveratrol, the equivalent amount of resveratrol content in DJ526 media; DJ indicates the diet in which 50% of cornmeal was replaced with the Dongjin callus; and DJ526 indicates the diet in which 50% of cornmeal was replaced with the DJ526 callus (Table S1). The body weights were measured on the 10th, 30th, 60th, and 90th day. The data are from three independent experiments, and values are shown as mean ± s.d. An unpaired Student’s *t*-test was used for the statistical analysis; *n* = 30, * *p* < 0.05, ** *p* < 0.01.

As increment of body weight is an important indicator of aging, the body weights of the DJ526-fed *D. melanogaster* were measured to evaluate the antiaging efficacy. During the whole experiment period, the body weights of flies, both ORR and Harwich, increased continuously (Figure 3). The flies of the DJ526 group, however, maintained a healthy body weight (Figure 3), as expected from its antiaging data on lifespan extension and amelioration of locomotive deterioration. The gross significances of the DJ526 group were observed starting from the 60th day. On the 90th day, the body weights of the DJ526 group of ORR flies were 1.79 ± 0.06 mg and 2.04 ± 0.06 mg for male and female, respectively, which were significantly lighter than the body weights of the DJ group (1.95 ± 0.03 mg and 2.18 ± 0.06 mg), RES group (2.01 ± 0.12 mg and 2.23 ± 0.08), and control group (1.96 ± 0.08 mg and 2.21 ± 0.11 mg). Similarly, on the same time point, the body weights of the DJ526-fed Harwich male and female flies were 2.13 ± 0.13 mg and 2.20 ± 0.05 mg, respectively, which were significantly lighter than the body weights of the DJ-fed group (2.54 ± 0.08 mg and 2.51 ± 0.02 mg), RES-fed group (2.64 ± 0.15 mg and 2.65 ± 0.08 mg), and control group (2.68 ± 0.11 mg and 2.67 ± 0.06). It should be noted that Harwich female flies on DJ diet were significantly heavier compared to the DJ526 diet during the ageing process (Figure 3D), although they had similar lifespan (Figure 1D) as the flies on the DJ526 diet. Overall, the resveratrol rice DJ526 callus exhibited health benefits, which was not observed in the other groups.

### 3.5. The Resveratrol-Enriched Rice DJ526 Callus Inhibited Eye Degeneration of D. melanogaster during Age Progression

**Figure 4:**
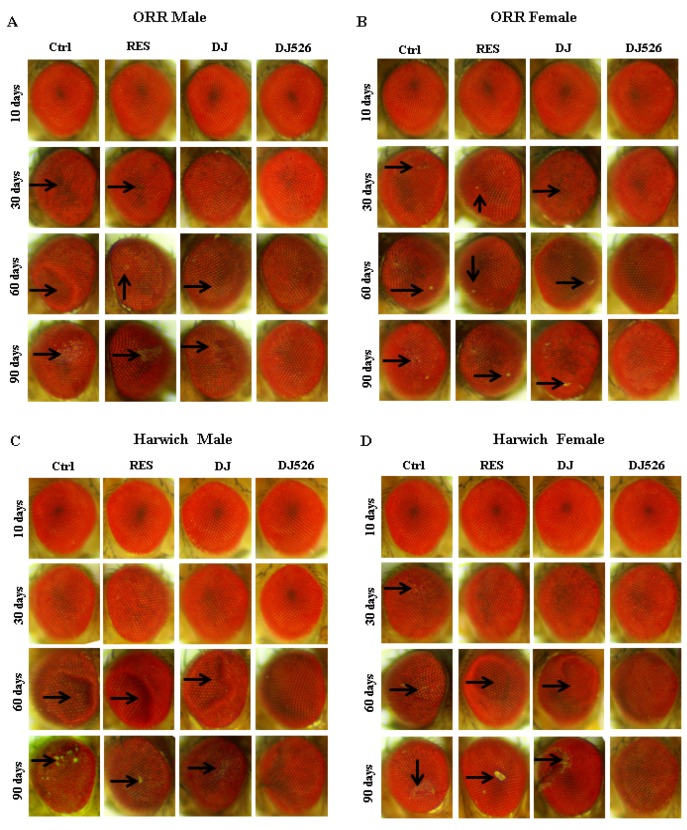
The resveratrol-enriched rice DJ526 callus inhibited the eye degeneration of *D. melanogaster* with age progression. (**A**) ORR male, (**B**) ORR female, (**C**) Harwich male, and (**D**) Harwich female. Ctrl indicates the standard cornmeal diet of *Drosophila*; RES (resveratrol) indicates the standard cornmeal diet supplemented with 5692.42 µg/L resveratrol, the equivalent amount of resveratrol content in DJ526 media; DJ indicates the diet in which 50% of cornmeal was replaced with the Dongjin callus; and DJ526 indicates the diet in which 50% of cornmeal was replaced with the DJ526 callus (Table S1). Light microscopic studies of the *Drosophila* compound eye of four experimental groups were conducted on the 10th, 30th, 60th and 90th day post-eclosion, and eye damages are indicated as arrows.

It has been reported that the *Drosophila* eye is undoubtedly a powerful system to detect any changes in tissue architecture and can be used as a tool to assess the combined effects of neurodegeneration as well as aging, thereby making *Drosophila* eye one of the most favored model for aging analysis [29]. Therefore, we assessed the morphological changes, including loss of eye pigment, eye damage, and roughness of *Drosophila* eye during age progression (Figure 4). Progressive loss of eye pigmentation and damage was observed upon age progression, starting from 30th day in some flies and 60th day in most flies. Interestingly, the eyes of both ORR and Harwich flies of the DJ526 group were rarely damaged even on the 90th day of the feeding experiments unlike the other three groups with age-dependent alterations of eye phenotype (Figure 4). These results indicated that the resveratrol rice DJ526 callus inhibited age-dependent eye degeneration upon aging.

### 3.6. The Resveratrol Rice DJ526 Callus Ameliorated Neurodegeneration of D. melanogaster during Age Progression

**Figure 5:**
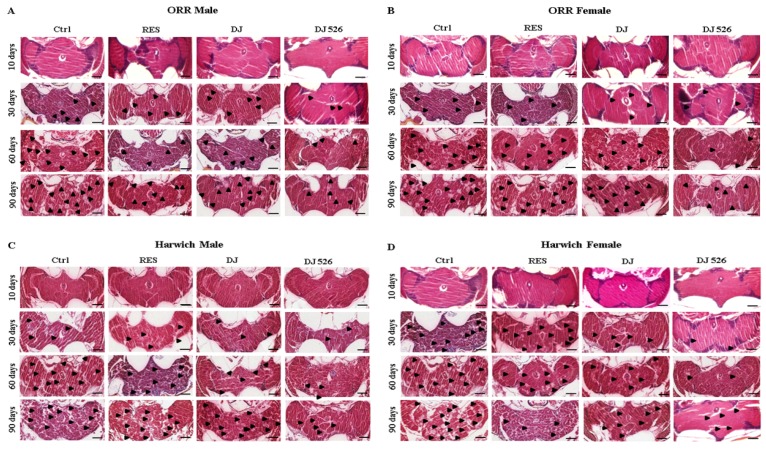
The resveratrol rice DJ526 callus ameliorated the neurodegeneration of *D. melanogaster* with age progression. (**A**) ORR male, (**B**) ORR female, (**C**) Harwich male, and (**D**) Harwich female. Ctrl indicates the standard cornmeal diet of *Drosophila*; RES (resveratrol) indicates the standard cornmeal diet supplemented with 5692.42 µg/L resveratrol, the equivalent amount of resveratrol content in DJ526 media; DJ indicates the diet in which 50% of cornmeal was replaced with the Dongjin callus; and DJ526 indicates the diet in which 50% of cornmeal was replaced with the DJ526 callus (Table S1). Histological analysis was carried out on the 10th, 30th, 60th and 90th day post-eclosion by hematoxylin and eosin (H&E) staining to observe the vacuole lesion and integrity of the *Drosophila* brains. Vacuole lesions are indicated as arrows. Scale bar, 200 μm.

**Figure 6:**
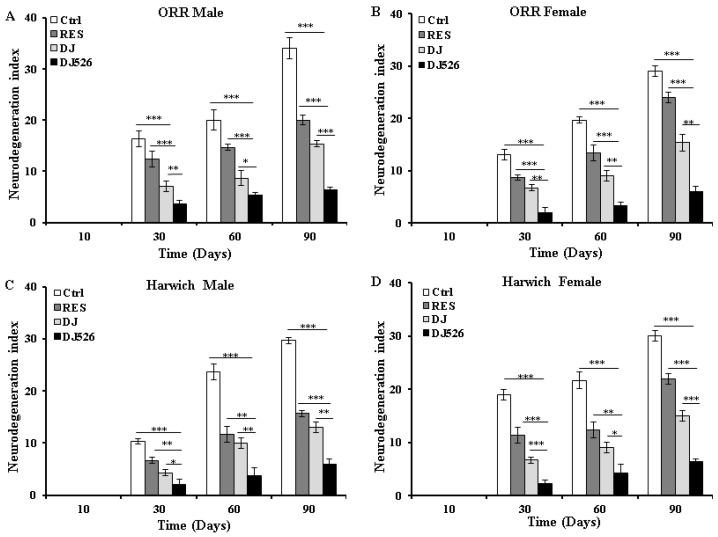
The resveratrol rice DJ526 callus prevented the age-related neurodegeneration of *D. melanogaster* with age progression. (**A**) ORR male, (**B**) ORR female, (**C**) Harwich male, and (**D**) Harwich female. Ctrl indicates the standard cornmeal diet of *Drosophila* RES (resveratrol) indicates the standard cornmeal diet supplemented with 5692.42 µg/L resveratrol, the equivalent amount of resveratrol content in DJ526 media; DJ indicates the diet in which 50% of cornmeal was replaced with the Dongjin callus; and DJ526 indicates the diet in which 50% of cornmeal was replaced with the DJ526 callus (Table S1). The quantification of neurodegeneration based on the histological analysis in the brains of *Drosophila* was observed on the 10th, 30th, 60th and 90th day post-eclosion. Data are from three independent experiments, and values are shown as mean ± s.d. An unpaired Student’s *t*-test was used for the statistical analysis; *n* = 100, * *p* < 0.05, ** *p* < 0.01, *** *p* < 0.001.

Because all of the above experiments showed that the resveratrol rice DJ526 callus ameliorated age-related symptoms as well as prevented aging of *D. melanogaster*, we investigated the efficacy of the resveratrol rice DJ526 callus on the brain tissue. We isolated the brain slices of each group of flies for H&E staining. As the appearance of vacuolar lesions in the brain slice is the major indicator of neurodegeneration [30], we examined all the H&E-stained slices of brains to determine the effect of the resveratrol rice DJ526 callus on age-dependent neurodegeneration of *D. melanogaster* (Figure S2, Figure 5; Figure 6). The DJ526 group maintained healthy brain integrity with significantly less quantity of vacuolar lesions, whereas vacuolar lesions appeared progressively and spread during the aging process in the RS and DJ groups as well as in the control group (Figure 5, Figure S2). The DJ526 group also demonstrated significant inhibition of neurodegeneration upon aging, while the RS and DJ groups showed mild amelioration compared to the control (Figure 6). Overall, the histological observation indicated that the resveratrol rice DJ526 callus efficiently inhibited age-related neurodegeneration.

## 4. Discussion

One of the most important and difficult current scientific challenges is to increase the lifespan of human beings. In this context, resveratrol has drawn significant scientific attention since the earlier observation about lifespan extension in yeast. The pro-longevity effect of resveratrol, however, has been challenged by many animal experiments in which resveratrol either failed to increase the lifespan or only had a marginal effect [15,19,21]. Furthermore, the therapeutic efficacy of pure resveratrol has shown mixed results in animal experiments, not only for longevity but also for other health benefits [15,21,31,32,33]. Despite the marginal effects of pure resveratrol, our previous studies showed that resveratrol rice DJ526 demonstrated unexpectedly high beneficial effects on metabolic syndrome and obesity through a synergistic mechanism [22,23,24,25]. In this work, we showed that the health benefits of resveratrol in the resveratrol rice DJ526 callus are not limited to metabolic syndrome and obesity. The DJ526 callus feed significantly extended the median lifespan of *D. melanogaster* by up to 50%, while simple addition of the same amount of resveratrol or the callus from the parent DJ rice failed to show the same degree of extension.

One of the key characteristics of multicellular organisms, such as high animals, is that the phenotypes of multicellular organisms are determined by the interaction of multiple genes rather than one gene. Recent findings have even added gut microbiota as a key determining factor for phenotypes of multicellular organisms in addition to genetic factors [34,35,36,37,38]. Considering these characteristics, it is not surprising to observe that synergistic treatment affecting multiple pathways dramatically boosts the original therapeutic nature of resveratrol in multicellular organisms. So far, various compounds promoting longevity have been identified, including resveratrol [5], rapamycin [6], metformin [7], spermidine [8], etc. However, these compounds have only demonstrated marginal extension of lifespan in wild-type animals. From this viewpoint, this work could provide an important clue for the development of an antiaging agent for humans.

Although the administration of resveratrol alone into *D. melanogaster* showed almost no effect in extending the lifespan (Figure 1), it slightly ameliorated age-related deterioration (Figure 2, Figure 3, Figure 4, Figure 5 and Figure 6). These results are consistent with previous findings, where pure resveratrol treatment showed no or insignificant effect in extending the lifespan of *D. melanogaster* [14,15]. Therefore, the synergistic effect of resveratrol in the resveratrol rice DJ526 will lead to insights into developing a therapeutic agent for longevity or age-dependent diseases. Furthermore, this work shows that exploring the synergistic interaction of bioactive chemicals or nutrients in vivo gives us new hope for the development of therapeutic agents to improve health as well as nutraceutical supplements for longevity.

## Supplementary Materials

The following are available online at https://www.mdpi.com/2072-6643/11/5/983/s1. Figure S1: A large amount of resveratrol production was achieved by the callus culture of the resveratrol rice DJ526, Figure S2: The resveratrol rice DJ526 callus decreased the number of vacuolar lesions in the brains of D. melanogaster with age progression, Figure S3: Scoring system is developed to quantify neurodegeneration, Table S1: The composition of the media in this study.

## References

[1] López-Otín C., Blasco M.A., Partridge L., Serrano M., Kroemer G. (2013). The hallmarks of aging. Cell.

[2] Vijg J., Campisi J. (2008). Puzzles, promises and a cure for ageing. Nature.

[3] Flatt T. (2012). A new definition of aging?. Front. Genet..

[4] Harman D. (1991). The aging process: Major risk factor for disease and death. Proc. Natl. Acad. Sci. USA.

[5] Fremont L. (2000). Biological effects of resveratrol. Life Sci..

[6] Harrison D.E., Strong R., Sharp Z.D., Nelson J.F., Astle C.M., Flurkey K., Nadon N.L., Wilkinson J.E., Frenkel K., Carter C.S. (2009). Rapamycin fed late in life extends lifespan in genetically heterogeneous mice. Nature.

[7] Martin-Montalvo A., Mercken E.M., Mitchell S.J., Palacios H.H., Mote P.L., Scheibye-Knudsen M., Gomes A.P., Ward T.M., Minor R.K., Blouin M.J. (2013). Metformin improves healthspan and lifespan in mice. Nat. Commun..

[8] Morselli E., Galluzzi L., Kepp O., Criollo A., Maiuri M.C., Tavernarakis N., Madeo F., Kroemer G. (2009). Autophagy mediates pharmacological lifespan extension by spermidine and resveratrol. Aging.

[9] Bhullar K.S., Hubbard B.P. (2015). Lifespan and healthspan extension by resveratrol. Biochem. Biophys. Acta.

[10] Markus M.A., Morris B.J. (2008). Resveratrol in prevention and treatment of common clinical conditions of aging. Clin. Int. Aging.

[11] Marchal J., Pifferi F., Aujard F. (2013). Resveratrol in mammals: Effects on aging biomarkers, age-related diseases, and life span. Ann. N. Y. Acad. Sci..

[12] Howitz K.T., Bitterman K.J., Cohen H.Y., Lamming D.W., Lavu S., Wood J.G., Zipkin R.E., Chung P., Kisielewski A., Zhang L.L. (2003). Small molecule activators of sirtuins extend saccharomyces cerevisiae lifespan. Nature.

[13] Wood J.G., Rogina B., Lavu S., Howitz K., Helfand S.L., Tatar M., Sinclair D. (2004). Sirtuin activators mimic caloric restriction and delay ageing in metazoans. Nature.

[14] Bass T.M., Weinkove D., Houthoofd K., Gems D., Partridge L. (2007). Effects of resveratrol on lifespan in Drosophila melanogaster and Caenorhabditis elegans. Mech. Ageing Dev..

[15] Staats S., Wagner A.E., Kowalewski B., Rieck F.T., Soukup S.T., Kulling S.E., Rimbach G. (2018). Dietary resveratrol does not affect life span, body composition, stress response, and longevity-related gene expression in drosophila melanogaster. Int. J. Mol. Sci..

[16] Valenzano D.R., Terzibasi E., Genade T., Cattaneo A., Domenici L., Cellerino A. (2006). Resveratrol prolongs lifespan and retards the onset of age-related markers in a short-lived vertebrate. Curr. Biol..

[17] Yu X., Li G. (2012). Effects of resveratrol on longevity, cognitive ability and aging-related histological markers in the annual fish nothobranchius guentheri. Exp. Gerontol..

[18] Genade T., Lang D.M. (2013). Resveratrol extends lifespan and preserves glia but not neurons of the nothobranchius guentheri optic tectum. Exp. Gerontol..

[19] Kim E., Ansell C.M., Dudycha J.L. (2014). Resveratrol and food effects on lifespan and reproduction in the model crustacean daphnia. J. Exp. Zool. Part A Ecol. Genet. Physiol..

[20] Baur J.A., Pearson K.J., Price N.L., Jamieson H.A., Lerin C., Kalra A., Prabhu V.V., Allard J.S., Lopez-Lluch G., Lewis K. (2006). Resveratrol improves health and survival of mice on a high-calorie diet. Nature.

[21] Pearson K.J., Baur J.A., Lewis K.N., Peshkin L., Price N.L., Labinskyy N., Swindell W.R., Kamara D., Minor R.K., Perez E. (2008). Resveratrol delays age-related deterioration and mimics transcriptional aspects of dietary restriction without extending life span. Cell. Metab..

[22] Baek S.H., Shin W.C., Ryu H.S., Lee D.W., Moon E., Seo C.S., Hwang E., Lee H.S., Ahn M.H., Jeon Y. (2013). Creation of resveratrol-enriched rice for the treatment of metabolic syndrome and related diseases. PLoS ONE.

[23] Baek S.H., Chung H.J., Lee H.K., D’Souza R., Jeon Y., Kim H.J., Kweon S.J., Hong S.T. (2014). Treatment of obesity with the resveratrol-enriched rice dj-526. Sci. Rep..

[24] Chung H.J., Sharma S.P., Kim H.J., Baek S.H., Hong S.T. (2016). The resveratrol-enriched rice dj526 boosts motor coordination and physical strength. Sci. Rep..

[25] Chung H.J., Lee H.K., Kim H.J., Baek S.H., Hong S.T. (2016). Gene expression profiles and physiological data from mice fed resveratrol-enriched rice DJ526. Sci. Data.

[26] White K.E., Humphrey D.M., Hirth F. (2010). The dopaminergic system in the aging brain of Drosophila. Front. Neurosci..

[27] Barone M.C., Bohmann D. (2013). Assessing neurodegenerative phenotypes in drosophila dopaminergic neurons by climbing assays and whole brain immunostaining. J. Vis. Exp..

[28] McPhee J.S., French D.P., Jackson D., Nazroo J., Pendleton N., Degens H. (2016). Physical activity in older age: Perspectives for healthy ageing and frailty. Biogerontology.

[29] Cutler T., Sarkar A., Moran M., Steffensmeier A., Puli O.R., Mancini G., Tare M., Gogia N., Singh A. (2015). Drosophila eye model to study neuroprotective role of creb binding protein (cbp) in alzheimer’s disease. PLoS ONE.

[30] Kounatidis I., Chtarbanova S., Cao Y., Hayne M., Jayanth D., Ganetzky B., Ligoxygakis P. (2017). Nf-κb immunity in the brain determines fly lifespan in healthy aging and age-related neurodegeneration. Cell. Rep..

[31] Bitterman J.L., Chung J.H. (2015). Metabolic effects of resveratrol: Addressing the controversies. Cell. Mol. Life Sci..

[32] Kaeberlein M., McDonagh T., Heltweg B., Hixon J., Westman E.A., Caldwell S.D., Napper A., Curtis R., DiStefano P.S., Fields S., Bedalov A. (2005). Substrate-specific activation of sirtuins by resveratrol. J. Biol. Chem..

[33] Guarente L., Picard F. (2005). Calorie restriction-the SIR2 connection. Cell.

[34] Liu J., Lkhagva E., Chung H.J., Kim H.J., Hong S.T. (2018). The pharmabiotic approach to treat hyperammonemia. Nutrients.

[35] Chung H.J., Nguyen T., Kim H.J., Hong S.T. (2018). Gut Microbiota as a missing link between nutrients and traits of human. Front. Microbiol..

[36] Nguyen T.T.B., Jin Y.Y., Chung H.J., Hong S.T. (2017). Pharmabiotics as an emerging medication for metabolic syndrome and its related diseases. Molecules.

[37] Chung H.J., Yu J.G., Liu M.J., Shen Y.F., Sharma S.P., Jamal M.A., Yoo J.H., Kim H.J., Hong S.T. (2016). Intestinal removal of free fatty acids from hosts by Lactobacilli for the treatment of obesity. FEBS Open Bio.

[38] Singh P., Chung H.J., Lee I.A., D’Souza R., Kim H.J., Hong S.T. (2018). Elucidation of the anti-hyperammonemic mechanism of Lactobacillus amylovorus JBD401 by comparative genomic analysis. BMC Genom..

