# Supplementary material for: The Resveratrol Rice DJ526 Callus Significantly Increases the Lifespan of Drosophila (Resveratrol Rice DJ526 Callus for Longevity)
Source: Nutrients. 2019 Apr 29;11(5):983. doi: 10.3390/nu11050983 (PMC6567216; doi:10.3390/nu11050983)
Supplement: Supplementary file 1 [file nutrients-11-00983-s001.pdf]

Supplementary Materials

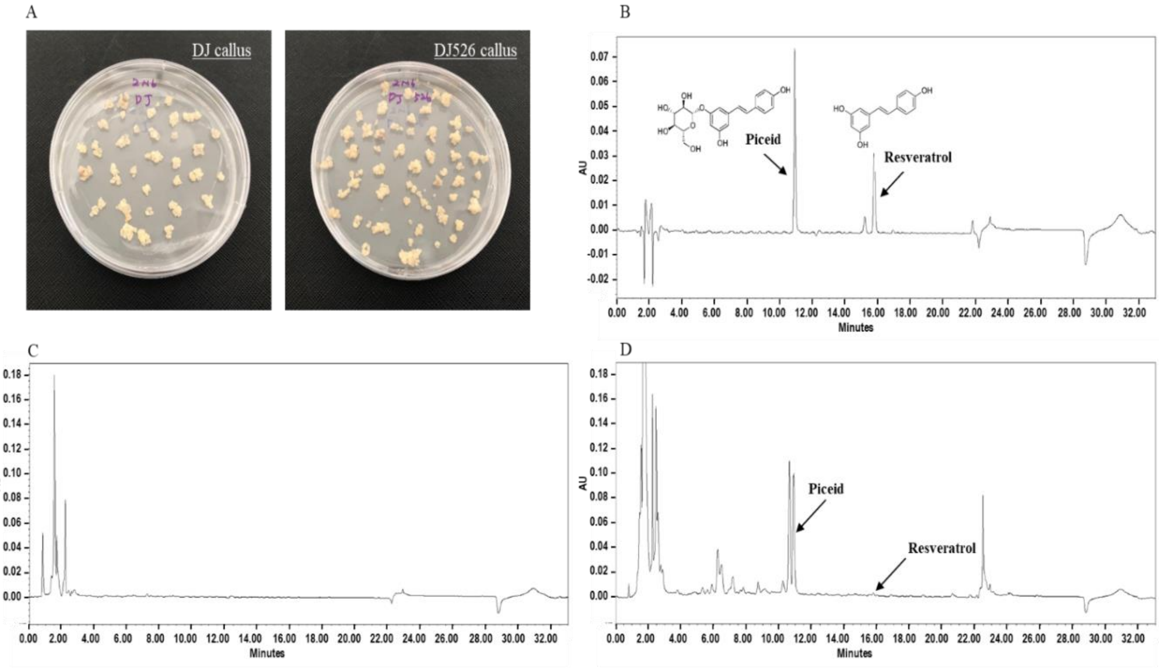

Figure S1. A large amount of resveratrol production was achieved by the callus culture of the resveratrol rice DJ526. **(A)** The resveratrol rice DJ526 callus was induced from the seeds of resveratrol rice DJ526 (DJ526) while DJ callus from Dongjin rice (DJ). **(B)** The HPLC chromatogram of the standards of resveratrol, piceid and resveratrol. The HPLC chromatogram of DJ callus **(C)** and HPLC chromatogram of DJ526 callus **(D).**

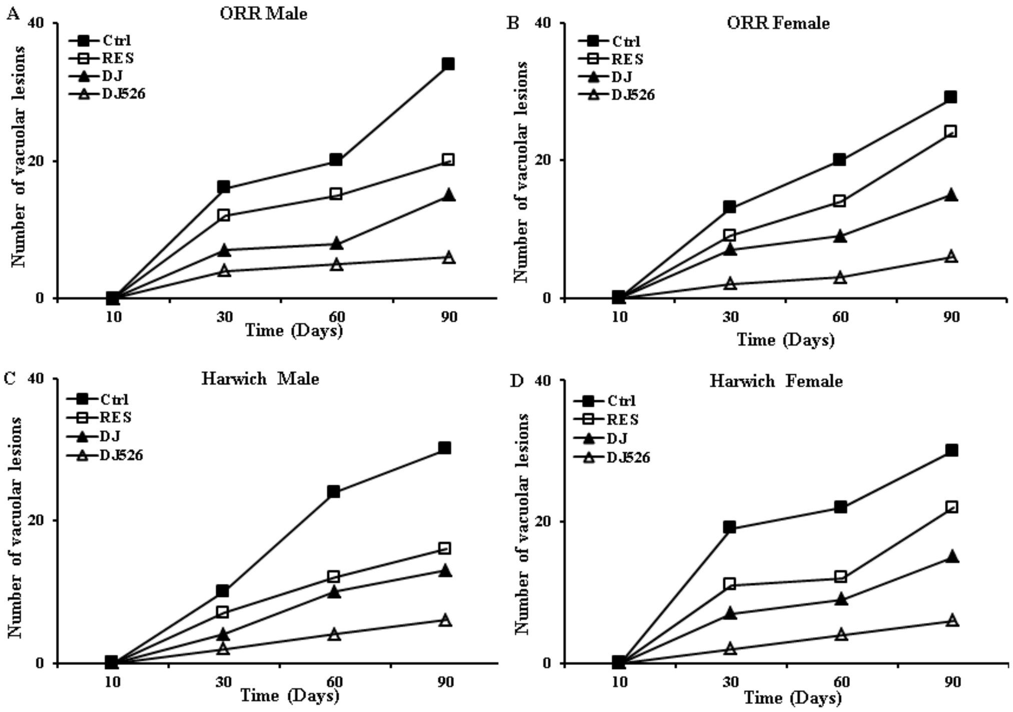

Figure S2. The resveratrol rice DJ526 callus decreased the number of vacuolar lesions in the brains of *D. melanogaster* with age progression. **(A)** ORR male, **(B)** ORR female, **(C)** Harwich male, and **(D)** Harwich female. Ctrl indicates the standard cornmeal diet of *Drosophila*; RES (resveratrol) indicates the standard cornmeal diet supplemented with 5,692.42 μg/L resveratrol, the equivalent amount of resveratrol content in DJ526 media; DJ indicates the diet in which 50% of cornmeal was replaced with the Dongjin callus; and DJ526 indicates the diet in which 50% of cornmeal was replaced with the DJ526 callus (Table S1). The quantification of vacuolar lesions, based on the histological analysis in the brains of *Drosophila* was observed at 10th, 30th, 60th and 90th days post-eclosion.

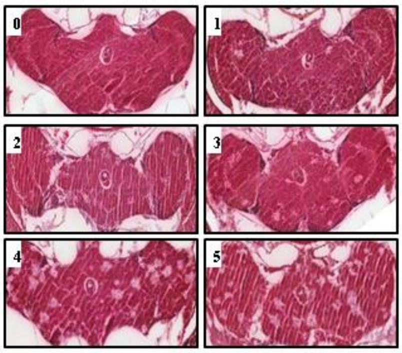

Figure S3. Scoring system is developed to quantify neurodegeneration. Each individual brain examined was scored from 0 to 5 based on the severity of the neurodegeneration by counting the number of vacuolar lesions as well as their sizes. Higher score indicates a more severe neurodegeneration. Representative paraffin sections corresponding to each score are given: 0, 1 normal to low; 2, 3 moderate; 4, 5 strong to severe.

Table S1. The composition of the media in this study.

Ingredient	Ctrl	RES	DJ	DJ526
Corn meal (g/L)	84	83.99	42	42
Yeast (g/L)	24	24	24	24
Sucrose (g/L)	47	47	47	47
Agar (g/L)	8	8	8	8
Molasses (ml/L)	25	25	25	25
10% Methyl perahydroxybenzoate (ml/L)	10	10	10	10
Propionic acid (ml/L)	4	4	4	4
Resveratrol (μg/L)	0	5692.42	0	0
DJ callus (g/L)	0	0	42	0
DJ526 callus (g/L)	0	0	0	42
